# Simultaneous multi-parametric acquisition and reconstruction techniques in cardiac magnetic resonance imaging: Basic concepts and status of clinical development

**DOI:** 10.3389/fcvm.2022.953823

**Published:** 2022-10-06

**Authors:** Katerina Eyre, Katherine Lindsay, Saad Razzaq, Michael Chetrit, Matthias Friedrich

**Affiliations:** ^1^McGill University Health Centre, Montreal, QC, Canada; ^2^Department of Experimental Medicine, McGill University, Montreal, QC, Canada

**Keywords:** cardiac MRI (CMR), undersampled acquisition, fast cardiac imaging, multiparametric cardiovascular magnetic resonance imaging, sub-Nyquist sampling

## Abstract

Simultaneous multi-parametric acquisition and reconstruction techniques (SMART) are gaining attention for their potential to overcome some of cardiovascular magnetic resonance imaging’s (CMR) clinical limitations. The major advantages of SMART lie within their ability to simultaneously capture multiple “features” such as cardiac motion, respiratory motion, T1/T2 relaxation. This review aims to summarize the overarching theory of SMART, describing key concepts that many of these techniques share to produce co-registered, high quality CMR images in less time and with less requirements for specialized personnel. Further, this review provides an overview of the recent developments in the field of SMART by describing how they work, the parameters they can acquire, their status of clinical testing and validation, and by providing examples for how their use can improve the current state of clinical CMR workflows. Many of the SMART are in early phases of development and testing, thus larger scale, controlled trials are needed to evaluate their use in clinical setting and with different cardiac pathologies.

## Introduction

Cardiovascular magnetic resonance imaging (CMR) is a versatile imaging modality that allows a quantitative assessment of cardiac function, morphology, blood flow, and tissue composition (1). A major advantage of CMR is its ability to directly characterize myocardial tissue without the need for invasive procedures or ionizing radiation (1, 2). While contrast agents are still frequently used, more and more techniques are now available that use native contrast mechanisms. The contrast in MR images arises primarily from variability in the proton density as well as longitudinal (T1) and transverse (T2) magnetic relaxation times of the tissue, which can be used to determine tissue composition based on quantitative T1 and T2 values (2). Since T1 and T2 values differ between different tissues and change with tissue pathologies such as inflammation or infiltration, T1 and T2 quantification strongly aids in differentiating between various cardiomyopathies including Fabry’s disease (3), amyloidosis (4), myocarditis (5), hypertrophic cardiomyopathy (6), takotsubo (7), or acute versus chronic ischemic cardiomyopathy (2, 8).

Although extremely informative, CMR is limited by its technical complexity and long acquisition times (9). Cardiac and respiratory motion make CMR particularly challenging. Images need to be acquired during a period where the patient is motionless, so the exam length heavily depends on the patients’ heart rhythm and on compliance with breathing instructions (10). Alongside patient compliance, scanning parameters must be carefully chosen with respect to pulse sequence type, spatial orientation of the imaging volume, and cardiac triggering options (10). These complexities demand specialized training of the medical staff and impair the clinical utility and accessibility of CMR, despite its widely accepted role in diagnosing cardiac disease (9).

To address these limitations, several working groups have focused on the development of fast and user-friendly acquisition methods (11–34). One proposed approach is the use of “one-click” scans, where multiple cardiac parameters (such as T1 relaxation, T2 relaxation, or cardiac motion) are collected simultaneously with less prospective planning (22, 23, 35, 36). These techniques have been collectively called Simultaneous Multiparametric Acquisition and Reconstruction Techniques (SMART) (37). SMART involves the collective acquisition of quantitative CMR contrast parameters (e.g., T1 and T2) which would normally be acquired separately in a clinical CMR setting. These new methods may increase sampling efficiency during free-breathing, ECG-free acquisitions and focus on retrospectively recovering data to reconstruct several cardiac contrasts at once. This may include producing simultaneous T1 and T2 maps, cine series or more (12, 14, 16, 18, 19, 23, 38–40). The result is a faster CMR acquisition with less need for specialized training, breathing instructions, or ECG setup. The goal of this review is to: (1) summarize the theory behind SMART, explaining how they enable the acquisition and reconstruction of high-quality images with less scan time compared to traditional methods; and (2) provide examples for how these rapid sequences could be applied in clinical settings, demonstrating how their application can improve the efficiency of clinical CMR scanning.

## Basics of multi-parametric sparse sampling methods

**FIGURE 1:**
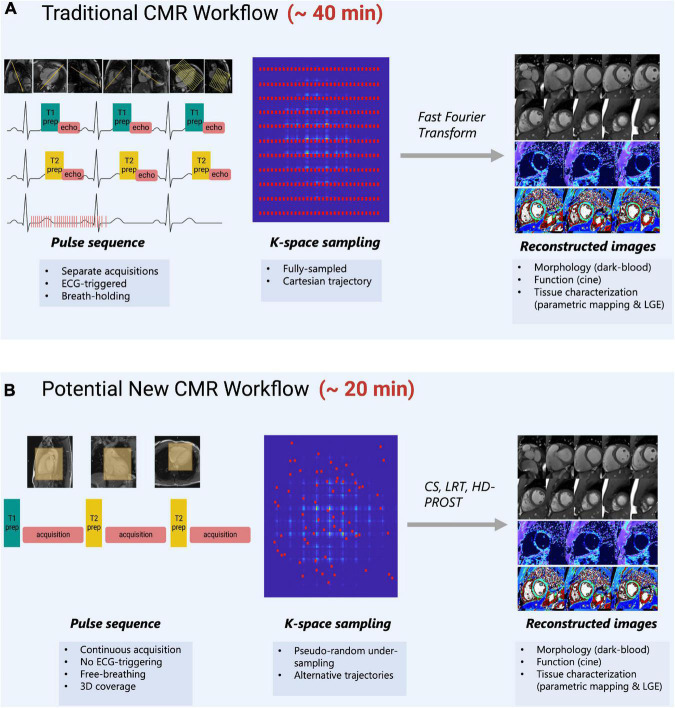
**(A)** Traditional CMR scanning workflow which requires separate image acquisitions to acquire images of different contrasts. Images are planned sequentially which requires time and specialized training to understand cardiac anatomy. ECG-triggers and breath-holding are needed to obtain images when motion is minimal. K-space is normally fully sampled and a fast-Fourier transform is used to obtain images with high image quality. **(B)** A potential new CMR workflow that is suggested by SMART-CMR. The novelty behind these SMART is to simplify CMR scanning by taking advantage of the redundancies which exist between images of different contrasts. Some SMART allow for imaging acquisitions without ECG-gating or breath-holding with whole-heart coverage. The acquisition planning is simplified, often simply requiring the placement of a volumetric box over the heart. For these methods to reduce scan time, pseudo-random under-sampling is often used in combination with alternative reconstruction approaches such as compressed-sensing (CS), low-rank tensor (LRT) methods, or high-dimensionality undersampled Patch based Reconstruction (HD-PROST).

Multi-parametric methods exploit the inherent redundancy of images to reduce the required sampling rate. Since redundant data can be compactly represented in some transform domains, this notion is closely related to the concept of “compressibility.” The redundancy that is present in conventional CMR acquisitions allows for reducing sampling rate requirements in SMART, resulting in decreased scan time. CMR’s long acquisition times are primarily caused by the need for several types of images−such as bright or dark blood morphological images, cine images, parametric maps, late gadolinium enhancement (LGE) images, perfusion images, or more−which require different parameter settings, views, and various contrast types (41). This limitation is worsened by the need for repeat measurements over various cardiac cycles to meet data sampling requirements and by the relatively short periods during which cardiac motion is minimal (10). There is significant redundancy with respect to anatomical regions being repetitively scanned for various contrasts (Figure 1A). The goal of SMART is to optimize the efficiency of CMR scans by acquiring multiple CMR data (cardiac motion, T1 relaxation, T2 relaxation etc.) in a single acquisition that can be reconstructed into informative images using assumptions based on prior knowledge of the MR signal properties (Figure 1B) (42).

**FIGURE 2:**
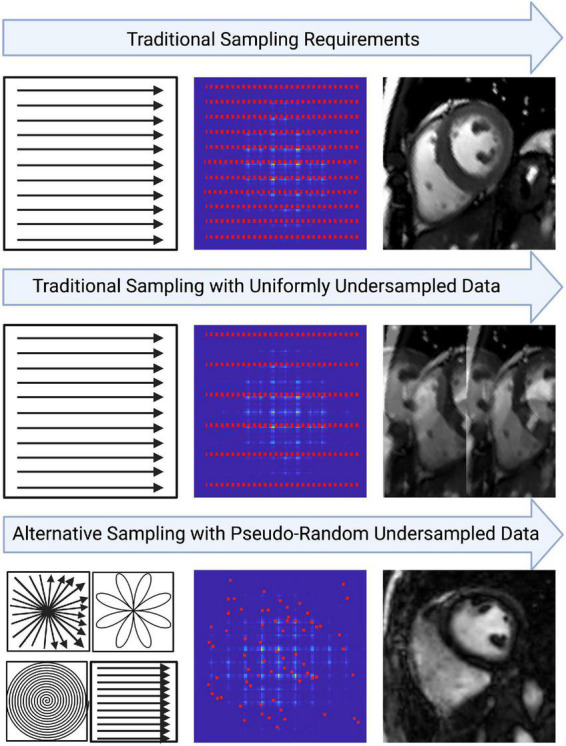
An example of how image quality may be affected from the way data is sampled in k-space. Traditional cartesian k-space sampling with fully sampled data results in high image quality. However, traditional k-space sampling with uniformly undersampled data results in coherent fold-over artifacts. Variable density pseudo-random undersampling allows artifacts to be incoherent in image space, approaching noise-like artifacts in some cases. This may allow the resulting image to retain diagnostic integrity despite heavy under-sampling. Oftentimes, variable density pseudo-random undersampling is achieved with alternative trajectories such as rosette, radial or spiral. The key benefits of these trajectories include the center of k-space being over-sampled which allows features like motion to be extracted and the incorporation of golden-angle sampling which strengthens the incoherence of artifacts in image space.

Sampling less MR data per reconstructed image typically results in reduced image quality (IQ), but this can be mitigated with alternative sampling trajectories (Figure 2) (42). Traditionally, MR data are acquired as signals on a Cartesian k-space grid and then reconstructed to an image using a Fast Fourier transformation (Figure 2) (10). When undersampled, this method leads to fold-over artifacts that may be detrimental to visual interpretation or quantitative analysis (Figure 2). Many newer techniques utilize alternative sampling trajectories which frequently sample through the center of k-space, such as radial, rosette or spiral trajectories. These trajectories are desirable because they allow detection and extraction of respiratory and cardiac motion, thus enabling newer techniques to be free-breathing or self-gated (35). This reduces scanning complexity for the technologist, as no ECG electrodes or respiratory navigators are required. Furthermore, it removes the need for monitoring breathing compliance, and eliminates the potential for cardiac mis-triggers or incomplete breath-holds. It also benefits pediatric patients or those who have difficulty holding their breath, and patients with abnormal cardiac rhythms.

So-called sparse reconstruction techniques can produce higher IQ with reduced scan time and comprehensive, co-registered images when they capture more information at once (42). Thus, three-dimensional (3D) acquisitions or those that simultaneously capture many features, such as varying MR contrasts, blood flow, and cardiac motion are characteristic of SMART. They effectively exploit the redundancy that exists in traditional CMR exams to achieve an efficient and nearly “all-in-one” image acquisition (42). This is also beneficial from a clinical standpoint as it allows the entire structure and function of the heart to be assessed with perfectly co-registered images across different MR contrast types.

Three methods, which are at the forefront of SMART, speed up MR acquisitions by undersampling (Table 1). These methods allow for recovery of CMR image integrity from data that were undersampled during their acquisition. The three key approaches are: Parallel Imaging (PI), Compressed Sensing (CS), and Low Rank Tensor (LRT) methods. These methods are commonly implemented alone or in combination.

**TABLE 1:** Comparison of acquisition and reconstruction properties of sparse sampling techniques discussed in this manuscript.

	Parallel imaging	Compressed sensing	Low rank tensor methods	HD-PROST
Trajectory	Any	Trajectories which allow incoherent aliasing	(1) Trajectories which allow incoherent aliasing (2) Trajectories which continuously sample low-frequency information (e.g., the center of k-space)	Trajectories which allow incoherent aliasing
Redundancy	Coil domain	Any sparsifying domain	Tensor representation	Tensor representation
Acceleration	2–3 fold	4–5 fold	4–5 fold	2.5–6.5 fold
Requirements	(1) Phased-array coils (2) Sensitivity maps	(1) Pseudo-random data sampling (2) Pre-selected “sparse” domain (3) Pre-selected number of tuneable parameters	(1) Multi-dimensional CMR acquisition (2) Pseudo-random data sampling (3) Formation of tensor	(1) Multi-contrast CMR acquisition (2) Pseudo-random data sampling (3) Formation of tensor
Assumptions	(1) Coils are most sensitive to the imaging-area they are closest to (2) Coil sensitivities vary throughout the image	(1) CMR data is compressible (2) Pseudo-random data sampling allows undersampling artifacts to be separated from “true signal” (3) Undersampled CMR data can be recovered in a “sparse” domain	(1) CMR data has many spatio-temporal-contrast correlations (2) Pseudo-random data sampling allows undersampling artifacts to be separated from “true signal” (3) High-dimensional CMR data can be expressed as a LRT (4) Undersampled CMR data can be recovered from a LRT model	(1) CMR data has many spatio-temporal-contrast correlations (2) Pseudo-random data sampling allows undersampling artifacts to be separated from “true signal” (3) A multi-contrast image can be expressed as a LRT (4) Joint-contrast, undersampled CMR data can be recovered from a LRT model
Adjustable parameters	(1) Acceleration factor (limited by number of phased-array coils) (2) SENSE vs. GRAPPA	(1) “Sparse” domain (2) Tuneable parameters in the reconstruction	(1) Tensor constraints (global vs. local) (2) Tuneable parameters in the reconstruction	(1) Tuneable parameters in the reconstruction
Clinical validation studies	(44, 112–115)	(30, 49–53, 57, 116–118)	(73, 84, 104)	(18, 19, 75, 87, 105)
Technical literature	(119–123)	(45–48, 54–56, 59, 60, 124, 125)	(23, 64, 66, 69–72, 74, 76–78, 83, 85, 103, 126, 127)	(25, 36, 86)

### Parallel imaging

**FIGURE 3:**
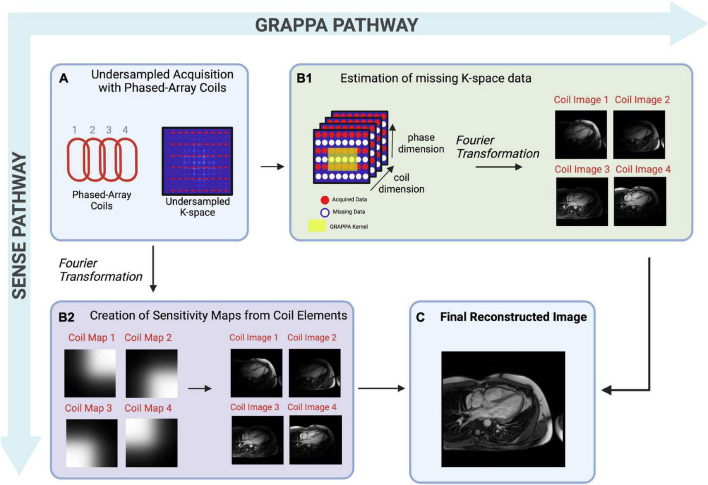
A pictorial example of multi-coil Parallel Imaging techniques describing both image-based SENSE (sensitivity encoding) or k-space based GRAPPA (generalized autocalibrating partial parallel acquisition)– two differing reconstruction techniques. **(A)** Pictorial representation of phased-array coils used to acquire undersampled data, and a graphical representation of undersampled k-space. The non-random undersampling method results in artifacts that are detrimental to visual inspection, therefore must be separated from real signals. **(B1)** Pictorial representation of k-space based GRAPPA. In this technique, reconstruction takes place in the k-space. The acquired MR signals fill in k-space for each coil, though many lines of k-space are missing due to undersampling. Missing points in k-space are then estimated iteratively using known data from the center of k-space, and local known data for each region, known as kernels. Once missing lines of k-space are filled, a Fourier transformation is performed, creating individual coil images from which the final reconstructed image will be created. **(B2)** Pictorial representation of image-based SENSE, in which reconstruction takes place in image space. A Fourier Transformation is performed which creates a coil sensitivity map for each coil element. These sensitivities are used to sort real signals from artifacts, creating an image for each coil, from which the final reconstructed image will be created. **(C)** The individual coil images are combined to create a final, reconstructed image.

Parallel imaging (PI) is widely used in clinical practice. PI allows for reduced data sampling by exploiting data redundancy available from phased array surface coils (Figure 3) (43). Phased array surface coils consist of several independent receiver coils arranged close to the region of interest (Figure 3). Each independent receiver coil is more sensitive to an anatomical area of the region of interest which is in closest proximity. Coil sensitivity maps are estimated and used to separate real signals from undersampling artifacts as the undersampled acquisition would typically lead to incoherent images if reconstructed using traditional methods (43).

Although PI improves the usability of CMR, scan time remains a significant limitation (9). While PI has allowed CMR scan time to be decreased 2- to 3-fold while maintaining diagnostic IQ (Table 1 and Figure 3) with shorter breath-hold times (44), it is limited by the fixed geometry of the phased array coil elements and the loss of SNR at greater accelerations, where less data are acquired (43). Greater field strengths and dimensionality increase the baseline signal, allowing for more coherent images despite acceleration (43). Thus, the benefits of PI are appreciated in CMR using higher magnetic field strengths and in exams that require a 3D or multi-dimensional component such as 2- or 3D cine imaging or angiography.

### Compressed sensing

**FIGURE 4:**
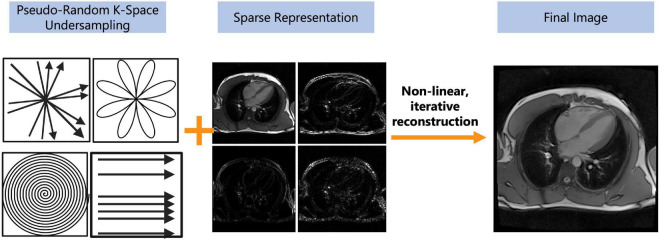
Depiction of the compressed sensing concept. Compressed sensing requires both a pseudo-random undersampling of k-space and for a sparse representation of the image in some transform domain (e.g., wavelet, finite difference, etc.). The pseudo-random sampling of k-space can be achieved with a variety of sampling trajectories such as radial, rosette, spiral, or cartesian. The under-sampled data undergoes a non-linear, iterative reconstruction to recover image integrity from the aliased image. The pseudo-random sampling of k-space allows undersampling artifacts to appear as noise and can be removed by thresholding in a sparse domain. The final image may have reduced image quality but should still be diagnostic.

Compressed sensing (CS) is a reconstruction technique that exploits the sparsity of an image to recover it from far fewer samples than required by the Nyquist–Shannon sampling theorem. To successfully reconstruct an image, CS requires the image to be sparse in some domain (e.g., wavelet, finite difference, etc.) and the undersampling artifacts to be incoherent in the sparse domain (Figure 4) (45). CS has enabled many applications, including removing the need for patient breath-holding in 2D (46) or 3D cine imaging (47), accelerating parametric mapping acquisitions (48), acquiring 3D LGE images (49, 50), acquiring 3D MR angiography images (51–55), or acquiring higher dimensional CMR images such as 5D cardiac images (x, y, z spatial dimensions + respiratory motion + cardiac motion) (30, 56–60). CS has also recently been cleared by the United States Food and Drug Administration (FDA), allowing it to be used and tested in larger clinical settings (61–63).

For CS to successfully reconstruct an undersampled image, data must be sampled pseudo-randomly, and the reconstruction process must iteratively threshold the sparse domain and enforce data consistency with the acquired data in k-space (the sampling domain) to separate signals from noise and undersampling artifacts (Figure 4) (45). A sparse domain is a domain where the image can be compressed, meaning that few signals are representative of the whole image (Figure 4). The undersampling artifacts must be “incoherent” in this domain, meaning that undersampling in k-space will not affect the detection of “real signals” in the sparse domain (45). Once an appropriate sparse domain is selected, data is sampled pseudo-randomly in k-space, with the sampling rate defined by a set acceleration factor (Figure 4). After sampling, the goal of the CS reconstruction algorithm is to recover the “true signal” and remove the aliased signal which appears as noise (Figure 4) (45). This is done using an iterative, non-linear reconstruction framework (45) (Figure 4).

Certain parameters can be altered within the CS framework to enhance the resulting IQ. Like PI, CS is more reliable when the baseline signal is higher, e.g., with higher field strengths or higher dimensional imaging (3D or more) (45). The choice of the “sparse domain” and sampling trajectory also impacts the robustness of the technique (45). An important parameter in the CS reconstruction called regularization strength increases image sparsity in the sparse domain and thus attenuates more noise but also results in spatial blurring, making detection of edges such as the myocardial blood pool border more difficult (45). Clinical studies to date have shown that CS can reduce scan times by up to 90% (Table 1) and increase patient comfort, without a significant loss of diagnostic IQ or information. Before widespread clinical adoption however, standardization of CS techniques is required (Figure 4).

### Low rank tensor methods

**FIGURE 5:**
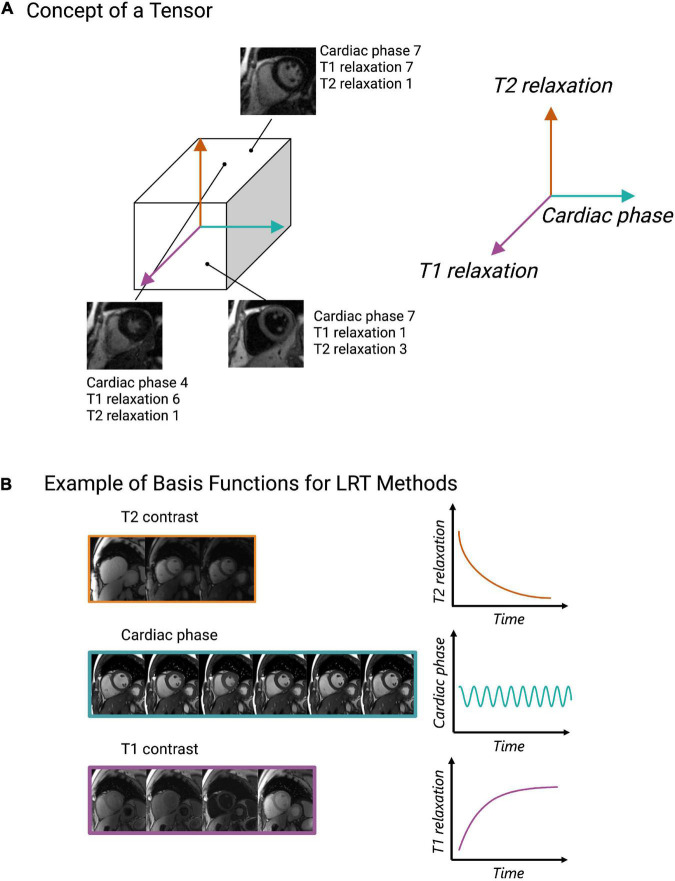
Depiction of the low rank tensor (LRT) concept. **(A)** Example of CMR images represented as a tensor with cardiac phase, T1 relaxation, and T2 relaxation representing different dimensions of the tensor. Once CMR data is organized in this way, any coordinate along the tensor will obtain an image with a various cardiac phase, T1 and T2 relaxation time. **(B)** Pictorial example of basis functions, which capture the signal behavior of each dimension (cardiac, T1 relaxation or T2 relaxation). These basis functions allow CMR data to be undersampled and for missing data to be recovered through various linear combinations of the sampled data.

Low rank tensor (LRT) methods are yet another way to exploit CMR redundancy and save scan time. This method frames CMR as a tensor (Figure 5A) and reduces the redundancies which exist within this tensor representation (64). For example, CMR data is a tensor when visualized as still frame images grouped by T1 relaxation, T2 relaxation, and cardiac phase (Figure 5A). When visualized in this way, anatomical, contrast, and signal overlap can be observed between frames (Figure 5A). LRT methods use correlations between frames to recover CMR images from undersampled data (65). In this sense, many MR features, such as respiratory or cardiac motion, can be viewed as higher dimensions within the LRT framework. This method has been applied to accelerate cardiac cine imaging (64, 66–71), visualization of contrast inflow (perfusion) (72), 5D flow (73), LGE (74), MR angiography (75), and parametric mapping (19, 25, 76). Like CS, LRT methods can be applied to remove the need for patient breath-holding or cardiac gating, making this another potentially useful method for difficult patient populations.

The key property that enables undersampling and scan time reduction in LRT methods is low rankness (64). Low rankness with respect to CMR means that along each dimension of the tensor (spatial, respiratory, cardiac, T1 relaxation, T2 relaxation, etc.), any datapoint can be obtained as a linear combination of other datapoints (64). In other words, a cardiac image at a specific cardiac phase with a specific contrast can be created by other images in different phases with different contrast weightings. The significance of this with respect to CMR scanning is that only a subset of CMR data is needed to extract higher-dimensional CMR data. However, to exploit these linear combinations, basis functions, i.e., functions which capture the signal behavior of each dimension (spatial, respiratory, cardiac, T1 relaxation, T2 relaxation, etc.), must be estimated (64) (Figure 5B). These can be estimated from the data itself or from Bloch equation simulations using the scan parameters (35).

**FIGURE 6:**
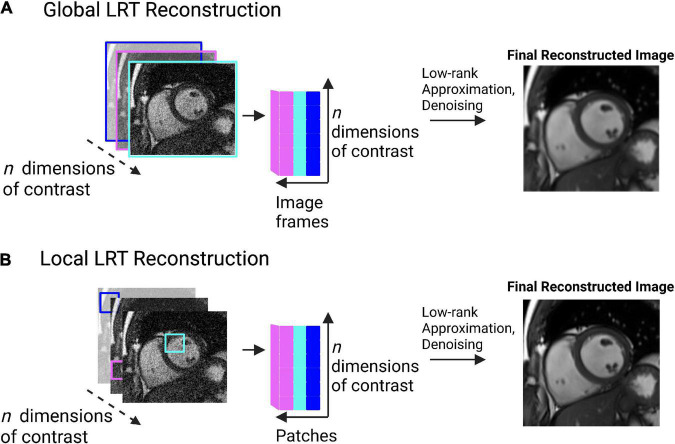
**(A)** Pictorial example of global low rank tensor (LRT) methods. This global method looks for image redundancy between entire image frames. This pictorial shows image frames identified across multiple image contrasts (e.g., T1, T2, etc.). Correlations are sought between the image frames, represented by the cartesian plane. Low-rank approximation and denoising are then applied to produce a final, reconstructed image. Spatial blurring or artifacts may be present in the resulting image due to the possibility of multiple tissue types being present in the image frame. **(B)** Pictorial example of local low rank tensor methods. This method breaks an image frame into “patches” and looks for image redundancy across image patches. The patches are unfolded in a matrix and a tensor is formed. Tensor decomposition through low-rank approximation allows for the image to be denoised, producing a final, reconstructed image. This method retains more detail information than the global method, as patches are more likely to contain a single tissue type, with a single contrast.

In LRT methods, two different perspectives can be used separately or jointly to exploit CMR redundancy: global or local (77). Using an example of 2D cine images, a global approach may look at each still frame as a whole and search for correlations between each image (Figure 6A). This will often result in residual artifacts or spatial blurring because of the many different contrasts (from fat, muscle, blood pool, and air) that are present in each frame (77). Global LRT treats different tissue types jointly, so the accuracy of defining fine details in an image is reduced (77). A local approach may break each still frame into smaller “patches” and search for correlations that exist between these patches across dimensions (Figure 6B) (77, 78). This method retains more of the image detail information because the patches are more likely to contain a single tissue type with a single contrast (77, 78).

A major advantage of LRT methods is that they are adaptive and versatile (35). Unlike CS, LRT methods do not require the selection of a pre-defined sparse domain; they can look at all the existing CMR data to find redundancy (35). The advantage of this is less dependence on *a priori* decision making and potentially a larger reduction of scan time with a greater retention of the MR signal. In imaging tasks where precision is important, such as with parametric mapping, LRT methods may be better suited than CS because less signal is lost during the reconstruction process (35, 78). Like PI and CS, LRT methods work best with higher dimensional CMR applications (3D or more) since more redundancy exists at higher dimensions. For this reason, LRT methods have been successfully applied to create joint T1-T2 or T1-T2-cine images (19, 23, 39, 79–85), exploiting the overlap between these contrasts. The adaptive and versatile nature of LRT methods have made them a major focus in the development of SMART (19, 23, 25, 36, 38, 39, 75, 79–86).

### High dimensionality, undersampled patch based reconstruction

**FIGURE 7:**
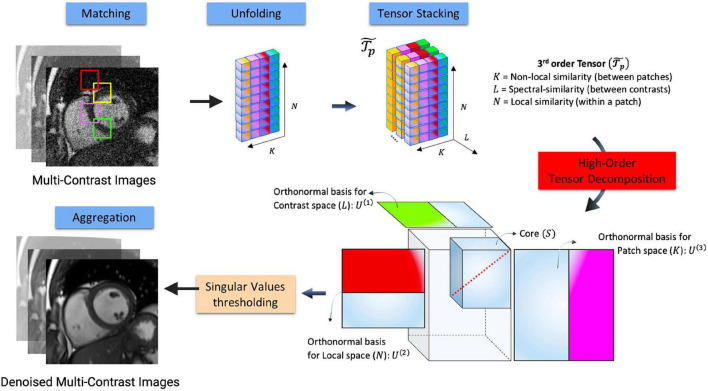
Flowchart describing the denoising HD-PROST optimization proposed by Bustin et al. (36). Multi-contrast images are denoised using 2D and 3D block matching, respectively, grouping similar 2D and 3D patches in the multi-contrast images. In a simple 2D matrix, these patches are then unfolded, and a third-order tensor is formed by stacking them in the contrast dimension. Through tensor decomposition, the high-order tensor can then be compressed. This is done by truncating multilinear singular vectors corresponding to small multilinear singular values. This process outputs denoised, multi-contrast images which are then used as prior knowledge in the joint MR reconstruction. Figure adapted from Bustin et al. (36).

High-dimensionality, undersampled patch-based reconstruction (HD-PROST) is a specific type of local-LRT regularization method which uses a patch-based perspective to exploit CMR redundancy (36) (Figure 7). Similar to local LRT methods, this patch-based approach breaks an image frame into “patches,” but unlike local LRT methods, it searches for correlations both within a given patch and between patches (36). This allows CMR redundancy to be exploited to an even greater extent than the aforementioned methods, translating to both a further reduction in scan time and production of higher-quality images (65, 74, 75).

To date, only proof-of-concept studies exist to demonstrate the clinical potential of applying HD-PROST using an undersampled MR acquisition (Table 2) (19, 25, 36, 38, 39, 75, 79–82, 86, 87). Recently, an HD-PROST application which recovers 3D cine, T1, and T2, was tested in phantoms and 10 healthy volunteers and gave comparable results for ejection fraction (EF) as well as highly precise T1 and T2 measurements when compared to standard methods (19). In another study, a free-breathing 3D whole-heart sequence capable of visualizing the coronary vasculature was used (75). Both phantom and *in vivo* images had an excellent agreement in visualizing the coronary vasculature and its distal segments when compared with the fully sampled reference image (75). These images had a good quality despite shorter scan times (4 min and 35 s ± 44 s vs. 22 min and 30 s ± 4 min and 54 s for fully sampled image) (75). HD-PROST has also been applied to reconstruct water- and fat-suppressed LGE images (87). Although this study found HD-PROST images to be of diagnostic quality in 18/20 datasets with strong agreement in the location of enhancement when compared to standard LGE images, residual cardiac motion was still present (87). This may be due to over-regularization causing mismatches in patch-similarity, producing noisy signal variations that are like aliasing artifacts (36). Thus, further clinical studies are needed to help standardize the tuning of hyper-parameters for cardiac applications that intend to use HD-PROST or any of the sparse-sampling methods previously described.

**TABLE 2:** Comparison of SMART methods discussed in this manuscript.

	Motion compensation method	Parameters acquired	Recon. schema	Acquisition schema (trajectory, prep. pulse type and readout)	Scan time	Recon. rime
Akçakaya et al. (11)	Breath held and ECG triggered	T1, T2	Voxel-wise least squares curve fitting	Cartesian trajectory with SR pulse and T2 prep pulse and a single-shot bSSFP readout	13 heartbeats	NA
Blume et al. (12)	Navigator-gated and ECG triggered	T1, T2	PI (1.6x acceleration) and least squares curve fitting	Cartesian trajectory with IR pulse and T2 prep pulse and bSSFP readout	NA	NA
Guo et al. (14)	Navigator-gated and ECG triggered	3D, T1, T2	Curve fitting by Levenberg-Marquardt algorithms	Cartesian trajectory with SR pulse and T2 prep pulse and GRE readout	7.9 ± 1.4 min	NA
Multi-mapping (15)	Breath held and ECG triggered	T1, T2	PI (2x acceleration) and Dictionary generation and matching	Cartesian trajectory with IR pulse and T2 prep pulse and bSSFP readout	NA	NA
SATURN (16)	Navigator-gated and ECG triggered	T1, T2, T2*	PI (3 or 4x acceleration) and curve fitting	Cartesian trajectory with SR pulse and T2 prep pulse and spoiled GRE readout	18.5 s/slice	NA
3D-QALAS (17)	Breath held and ECG triggered	3D, T1, T2	PI (2x acceleration) and curve fitting	Cartesian trajectory with IR pulse and T2 prep pulse and GRE readout	15 heartbeats	NA
Milotta et al. (18)	Navigator-triggered retrospectively and ECG triggered	3D, T1, T2, water, fat fraction	PI (4x acceleration), HD-PROST, motion correction, and dictionary generation and matching	Cartesian trajectory with spiral-like profile order, IR pulse, T2 prep pulse, Dixon GRE acquisition	9 ± 1 min 48 s	27 min and 45 s
Qi et al. (19)	Free breathing, non-ECG gated	3D, T1, T2, cine	PI (2x acceleration), cardiac/respiratory binning, HD-PROST, dictionary generation and matching	Radial (golden angle) trajectory with IR pulse and T2 prep pulse and spoiled GRE readout	11.2 min	NA
CABIRIA (20)	Breath held and ECG triggered	T1, T2	PI (2x acceleration), and curve fitting	Cartesian trajectory with IR pulse and bSSFP readout	8 heartbeats/slice	NA
Deep-BLESS (21)	Breath held and ECG triggered	T1, T2	DL algorithm, PI (2x acceleration), CS	Radial trajectory (golden angle) with IR pulse and T2 prep pulse and spoiled GRE readout	11 heartbeats/slice	<1 s
Finger- printing (22)	Breath held and ECG triggered (newer adaptations are free-breathing and non-ECG triggered) (38, 39, 101)	3D, T1, T2, T2*, ECV, proton density, cine, fat fraction, water, T1 rho (22, 39, 81, 82, 95, 96)	Different frameworks have been proposed: all use dictionary generation and matching; some additionally incorporate PI, CS, LRT, HD-PROST (38, 79–82)	Spiral trajectory with IR pulse and T2 prep pulse (newer adaptations use radial or rosette trajectories)	Various acquisition times depending on sequence (16 heartbeats/slice (22), 7 min (38), 29.4 s/slice (39)	NA
Multi-tasking (23)	Free breathing and non-ECG gated	3D, T1, T2, T2*, ECV, fat fraction, cine	Cardiac/respiratory binning, PI, LRT	Radial trajectory with a hybrid T2IR preparatory pulse and GRE readout, and self-navigation with adequate temporal resolution to estimate motion basis-functions		

## Simultaneous multi-parametric acquisition and reconstruction techniques

Here, the SMART, which allow for simultaneously acquiring and reconstructing co-registered CMR images of different contrast weightings, with sparse sampling principles applied alone or in combination, are described.

### 3D single-parameter mapping

Some multi-parametric methods have focused on obtaining a single quantitative image contrast as part of a 3D or multi-cardiac-phase acquisition to increase the efficiency of parametric mapping CMR exams. Clinical CMR parametric mapping is limited by incomplete spatial coverage of the heart which decreases its sensitivity to detect regional myocardial abnormalities (37). Given the good agreement of mapping with LGE enhancement in visualizing focal lesions (88), complete coverage would likely increase the sensitivity of mapping and may even allow for avoiding contrast agent administration altogether.

In recent years, several groups have presented methods to obtain whole-heart T1 maps during a free-breathing acquisition. Han et al.’s (29) method exploits redundancy in the temporal domain (e.g., between image frames) to reduce scan time and obtain 40 short-axis slices with a spatial resolution of 1.9 × 1.9 × 4.5 mm. Their method requires a fairly lengthy imaging time of 14 min, but this time could be decreased by decreasing the number of slices acquired or lowering the spatial resolution (29). Nordio et al. (89) similarly developed a free-breathing 3D T1 mapping method which obtains 11 short-axis slices in 12 min. Their method incorporates an image-denoising step before T1 map fitting which improved the precision of their T1 maps when compared to Modified-Look-Locker-Inversion-Recovery (MOLLI) (89). However, their method depends on a 1D respiratory navigator which was shown to achieve a scan efficiency of only 36% when tested in 15 healthy subjects (89). In 2020, Guo et al. (90) presented a free-breathing 3D T1 mapping method which obtains nine short-axis slices in only 2 min. This method achieved high precision of T1 mapping values when compared to both Saturation recovery single-shot acquisition (SASHA) and MOLLI methods (coefficient of variations: 6.2 ±1.4%, 5.3 ± 1.1%, and 4.9 ± 0.8% for SASHA, MOLLI and the proposed 3D method, respectively) (90) in a highly accelerated scan time, demonstrating clinical feasibility of the technique.

Recently, Bustin et al. (25) proposed a free-breathing technique, 3D Motion-Corrected Undersampled Signal matched (MUST)-T2, to obtain high spatial resolution (1.5 mm^3^) 3D T2 maps in 8 min. Their method, which is similar to Ding et al.’s (26), uses a saturation pulse to reset the magnetization after every heart beat to increase scan efficiency and reduce the dependence on heart rate (25). When tested in a cohort of 25 patients with myocarditis, the method demonstrated a high sensitivity to detect edema (25). The isotropic spatial resolution is advantageous because it allows reformatting in any imaging view without a loss of resolution. Van Heeswijk et al. (33) developed a similar 3D T2-mapping approach with isotropic resolution (1.7 mm^3^) but their method is slightly less efficient as a three-heartbeat waiting period is needed between magnetization recovery (scan time ≈ 18 min) (33). Milotta et al. (31) propose a similar technique which can obtain whole-heart T2 maps, dark- and bright-blood images in a free-breathing scan of 11 min with high spatial resolution. The 3D coverage and high-spatial resolution of the aforementioned methods may allow detection of the coronary arteries in addition to myocardial tissue characterization and morphological assessment, moving towards the direction of a comprehensive CMR exam.

### Joint T1–T2

In traditional T1 and T2 parametric mapping, a preparatory pulse [inversion recovery (IR), saturation recovery (SR), combination of IR and SR, or T2 preparatory (T2prep)] is used before a readout with a pulse sequence to generate a single contrast. These readouts occur at several different time points after the preparatory pulse is applied, and later each voxel’s signal intensity from the image series is fitted to a curve that describes the relaxation rate of the voxel (2). The acquisition is typically ECG-triggered with a breath-hold requirement for each obtained 2D slice. This method is not only lengthy but also depends on two major assumptions: (1) the voxels’ signal intensity can be described only by the relaxation time being measured (or that the influence of other factors are negligible), and (2) the images in the series are co-registered (i.e., there is no physical displacement between the voxels of images acquired at different readout time points) (2). Since T1 and T2 provide complementary information when characterizing myocardial tissue (2), joint T1–T2 mapping may both overcome lengthy acquisition times and increase the diagnostic utility of parametric mapping by providing co-registered maps.

To simultaneously generate T1 and T2 maps, a combination of IR or SR and T2 prep pulses are typically used to generate T1 and T2 contrasts, respectively (Table 2) (35). Blume et al. (12) presented one of the first joint T1–T2 techniques which could acquire joint images using IR and T2prep pulses in an ECG-triggered, navigator-gated, free-breathing acquisition. Although their method was shown to measure precise T1 and T2 values in 19 healthy subjects, their method is inefficient−requiring almost 3 min to obtain a single 2D slice−as it requires dummy heartbeats during signal recovery (12). Guo et al. (14) and Hermann et al. (16) also presented navigator-gated, free-breathing approaches to obtain joint maps. Guo et al.’s (14) method was shown to be relatively fast, acquiring 3D joint T1–T2 maps with an average scan time of 8 min with moderate precision (coefficient of variations: 6.0 for T1 and 10.6 for T2). Hermann et al. (16) generated T2* maps in addition to T1 and T2, using an average acquisition time of 26.5 s/slice. Although these methods were shown to rapidly acquire joint maps, it should be noted that their techniques were tested mainly in a healthy subject population where breathing is relatively consistent. Navigator-based triggering depends on a steady breathing pattern which may not be found in all patients and could result in increased scan times in clinical settings.

In a different approach to navigator-triggering, Milotta et al. (18) acquired 2D low-resolution image navigators before running their 3D sequence to retrospectively isolate respiratory motion. The 2D image navigators are acquired rapidly and simplify the acquisition as they remove dependence on obtaining optimal respiratory-triggering windows, but they neglect to consider breathing motion in the anterior-posterior direction which may impact the robustness of the mapping technique (18). Their method additionally incorporates the HD-PROST framework to increase scanning efficiency, obtaining joint T1–T2 and water-fat maps over the whole heart with isotropic resolution in just 9 min (18). Qi et al. (19) take this one step further by removing the need for respiratory navigators altogether through use of a radial sampling schema. During reconstruction, the breathing motion is estimated using the k-space center of all radial spokes (19). Their method obtains 3D joint T1–T2-cine maps with isotropic resolution in 11.2 min (19).

Kvernby et al. (17) present another IR and T2prep technique, 3D-QALAS, which can acquire a stack of 13 2D short axis co-registered T1–T2 maps at end-diastole in an ECG-triggered acquisition of 15 heartbeats. This method was shown to detect T1 and T2 changes in a longitudinal study of patients who underwent valve replacement surgery (91) and was highly reproducible in a cohort of 23 patients with mixed pathologies, although it suffers from a lower precision compared to MOLLI and T2-Gradient-Spin-Echo (GraSE) techniques (92). CABIRIA, which similarly acquires joint 2D T1/T2 mapping images in a breath-hold of eight heartbeats, achieved high precision when tested in five healthy subjects but suffered from low repeatability (20). Their method continuously acquires data throughout eight cardiac cycles after a user-selectable timepoint after the R-wave in the ECG (20). This has the advantage of increasing the efficiency of data collection, ultimately reducing acquisition time. In contrast to the combination of IR and T2prep pulses, Akçakaya et al. (11) used a combination of SR and T2prep pulses to generate 2D joint T1–T2 maps in a breath-hold of 13 heartbeats. Their method yielded improved T1/T2 accuracy but with lower precision compared to the IR based methods (11).

Other joint T1–T2 mapping approaches include Henningsson’s (15). Multimapping, deep learning (DL) Bloch equation simulations (DeepBLESS) (21, 93), and Chow et al.’s (13) mSASHA. Multimapping is a joint T1/T2 method which generates a dictionary for each subject and then matches the acquired signal to this dictionary to generate T1–T2 maps (15). They only partially resolve these dictionaries in order to reduce the otherwise lengthy dictionary generation process (15). DeepBLESS is based on the Bloch equation simulations with the slice profile correction (BLESSPC) algorithm which was previously developed for MOLLI T1 mapping (21, 93, 94). mSASHA uses an ECG-trigger to acquire joint T1–T2 maps in 11 heartbeats. Their method demonstrated both high accuracy and precision when tested in 10 healthy subjects.

All aforementioned methods address key challenges of CMR, including the complexity of CMR scanning procedures and long scan times, but are limited to small sample sizes in mostly healthy subjects. Thus, despite the growing body of evidence, further clinical validation, feasibility, accuracy, and impact-on-outcome studies are required to verify the clinical potential of these SMART.

### Cardiac magnetic resonance fingerprinting

**FIGURE 8:**
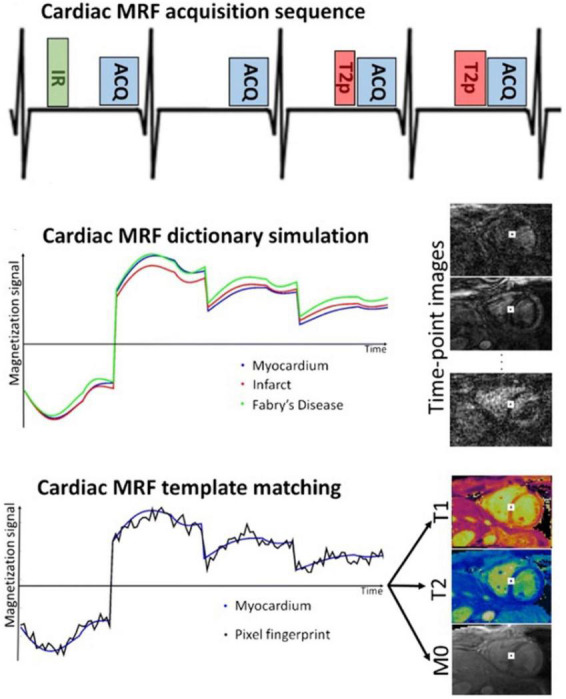
Example of the Fingerprinting workflow, including the dictionary matching process, and images obtained using the cardiac Fingerprinting sequence. This sequence simultaneously quantifies T1 and T2 in the myocardium. The figure shows acquisition of only 4-heartbeats of the 16-heartbeat acquisition method. In each heartbeat an inversion recovery (IR), T2 preparation (T2p), or no preparation pulse is used before the data acquisition (ACQ). Image taken from Cruz et al. (99).

Cardiac magnetic resonance fingerprinting (cMRF) is a multi-parametric, rapid acquisition sequence for simultaneous acquisition of multiple quantitative tissue parameters. The traditional cMRF sequence quantifies T1, T2, and proton density (M0) using an ECG-triggered sequence with a breath-hold of 16 heartbeats (Figure 8) (22). Several variants of the sequence have been added over time, among them modifications for detecting and quantifying T1p, T2*, and fat signal fraction (81, 82, 95, 96). Other developments include correcting for the confounding factors caused by the radiofrequency field (B1) (97), incorporating the ability for cMRF to acquire multiple cardiac slices at once (80), incorporating a 3D free-breathing, non-cardiac gated sequence with an acquisition time of 7 min (38) and more recently, incorporating a 2D joint T1–T2-cine sequence (Table 2) (39, 98).

Cardiac magnetic resonance fingerprinting attempts to capture the continuous and transient state of the magnetization history using various pulse sequence modules (e.g., IR, SR, T2prep pulses, varying flip angles and varying TR) that are sensitized to parameters of interest (e.g., T1 and T2) (22). The acquired signals are matched to a dictionary of possible signal evolutions to generate quantitative maps of interest (Figure 8) (22). cMRF dictionaries are generated using Bloch equations − a mathematical formula that calculates magnetization as a function of time with respect to T1 and T2 relaxation rates, and any other properties that can be modeled by the MR physics − to predict a range of possible spin behaviors and signal evolutions (22). In cardiac-triggered cMRF, dictionaries are made for each patient at the time of reconstruction, based on patient-specific patterns. They account for hardware parameters (B1 field inhomogeneity), acquisition parameters (pulse sequence type, echo time, flip angle, repetition time, readout type, etc.) and heart rate (22). This is critical as clinical parametric mapping is inherently susceptible to external factors (2). For a more thorough overview of the cMRF technique and its applications, the reader is directed to (98–100).

To save acquisition time, cMRF heavily undersamples data using radial, rosette, or spiral sampling trajectories (22). Radial or spiral trajectories are chosen so that the undersampling artifacts are incoherent in the spatial domain. Although each individual image is heavily undersampled, cMRF acquires hundreds of these poor-quality images, so that tissue signal patterns can still be identified and matched to the ‘fingerprint’ from the dictionary. More recently, cMRF has incorporated CS, LRT or HD-PROST methods into its framework (39, 79–82, 98). LRT methods have been applied to the original cardiac-gated and breath-held cMRF technique to acquire multiple 2D T1–T2 slices simultaneously and to acquire joint T2–T2-cine images (80, 98). HD-PROST has similarly been applied to obtain joint T1–T2-cine images and 3D free-breathing and non-cardiac-gated cMRF images (38, 39). Recent clinical trials have demonstrated that cMRF gives highly reproducible T1 and T2 measurements that correlate well with standard mapping sequences (Table 2). The main advantage of combining undersampling methods with existing SMART methods is to recover images with higher IQ and greater image detail due to their effective recovery of undersampled data (98–100).

Though many studies have demonstrated the clinical potential of cMRF (99), there is still a need for larger, prospective clinical trials to validate the technique for clinical application. To date, the largest study consists of 58 healthy volunteers scanned at a single site on a 1.5T scanner (101). The aim of this study was to test the precision, repeatability, and IQ of cMRF maps compared to standard mapping techniques. The authors found that though cMRF measurements were slightly less precise than conventional sequences, they were reliable and cMRF images showed a more consistent IQ compared to conventional sequences (101). Recently, cMRF was also tested in a cohort of nine patients with amyloidosis and was found to achieve high diagnostic accuracy of amyloidosis detection (102). This study was the first to test the technique in a controlled clinical trial but with a small sample size. Future trials should include different patient populations to bring the cMRF technique into clinical practice and to help focus the optimization of cMRF developments.

The quantitative nature of cMRF lends itself well to CMR protocols for myocardial tissue characterization. Parametric mapping such as T1 and T2 are important for differentiating between edema, scar, fatty tissue, and other abnormalities such as the deposition of amyloid fibrils (2). The simultaneous acquisition of multiple contrasts and maps in cMRF reduces error caused by a mismatch of anatomical positions or the cardiac phase across different image types.

### Cardiac magnetic resonance multitasking

**FIGURE 9:**
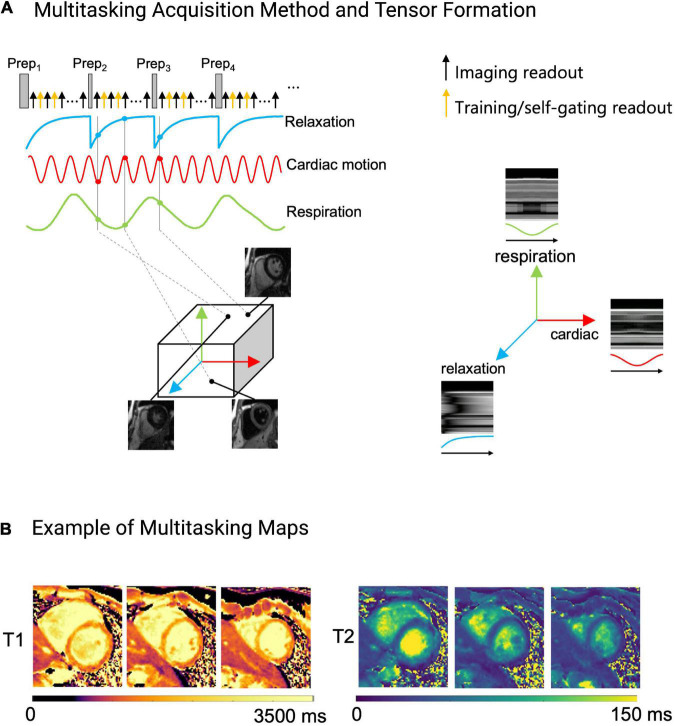
Example of the Multitasking acquisition method. **(A)** To capture T1 and T2 contrasts simultaneously, a hybrid T1/T2prep pulse is used in a continuous acquisition. Training/self-gating readouts are also acquired for retrospective cardiac and respiratory binning, and to estimate basis functions. Multi-dimensional information, namely cardiac and respiratory motion, and relaxation rate are extracted from the acquired data and sorted into a tensor. Each dimension in the tensor depicts cardiac motion, respiratory motion, T1 relaxation or T2 relaxation. One can choose any point within the tensor to obtain an image at a specific respiratory position, cardiac position, and relaxation position. **(B)** Example of the fitted Multitasking T1 and T2 maps. These maps are fitted after data are sorted into their tensor formation.

Magnetic resonance multitasking is a free breathing multi-parametric sequence that can resolve cardiac motion, respiratory motion and myocardial relaxation properties, without the need for ECG triggering (Figure 9) (23). Its reconstruction framework allows for the incorporation of PI, CS, and LRT methods to decrease scan time (23). This allows a patient to lay-down and breath normally while the standard acquisition of cardiac cine, T1 and T2 mapping is acquired as opposed to the traditional breath-held and ECG triggered methods. Different variants of Multitasking sequences exist with varying acquisition times, but on average, Multitasking can acquire parameters in 1.5 min/slice or 15 min for a 3D ventricular stack, using a satisfactory in-plane spatial resolution of 1.4 × 1.4 × 8 mm (23, 103).

A previously published Multitasking acquisition scheme can be described as follows (23): T1 and T2 contrast data can be acquired simultaneously with radial sampling after five hybrid T2/IR preparation pulses, with a subset of k-space being sampled more frequently than the rest of k-space to obtain substantial temporal information for the retrospective cardiac and respiratory binning and to derive respiratory and cardiac subspaces used in the LRT reconstruction (Figure 9A). Binning refers to the retrospective data sorting into their motion states (respiratory and cardiac phases). The rest of the k-space is undersampled.

In the reconstruction process, Multitasking first sorts the motion states and then fits the dynamic image frames to T1 and T2 contrast weightings (23). The T1 and T2 maps are generated in a similar but slightly different fashion to cMRF. Multitasking generates a dictionary of T1 and T2 recovery curves using Bloch equations and then determines the T1 and T2 basis functions from this dictionary to perform a voxel-wise fitting of the acquired imaging signals (23). CS and LRT methods are then used to recover IQ from the undersampled dataset (23). Since images have been sampled across the entire T1/T2 recovery period, cine images can be created using many different variations of T1 and T2 contrast weighting. One can obtain dark-blood, bright-blood, T1-weighted, and T2-weighted cine series (Figure 9A) (23). If the recovery times for pericardium, fat, scar, edema, and myocardium are known, additional images can be created that either suppress or highlight these tissues—all from the same acquisition. This removes the need for specialized training by allowing a comprehensive exam to be obtained without ECG-gating and while the patient is breathing freely.

Several proof-of-concept studies have confirmed the versatility of the Multitasking framework (Table 2). Multitasking has been used to measure myocardial T1 and extracellular volumes (ECV) (104), myocardial T1 and T2 (23, 85), myocardial T1, T2, T2*, and fat-fraction (83) and carotid plaques and aortic strain in patients with thoracic aortic disease (84). These studies demonstrated that the Multitasking framework can produce high quality images with reproducible values that are in good agreement with reference values (Table 2). However, clinical studies with larger sample sizes are required to confirm these findings and validate this sequence for clinical use. While Multitasking has a strong clinical potential and can add additional contrasts such as T2*, its development is still ongoing, and more pre-clinical and clinical studies have yet to show its ability to reliably quantify cardiac function, or T1 and T2 values in clinical settings.

## Deep learning applications to simultaneous multi-parametric acquisition and reconstruction techniques

**FIGURE 10:**
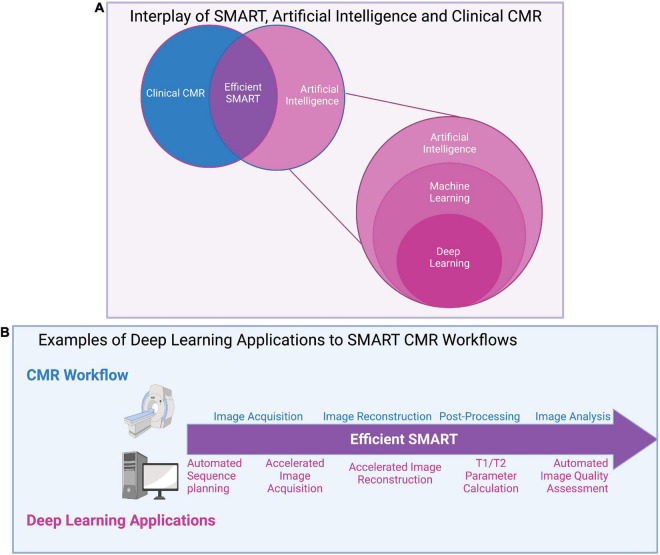
**(A)** Interplay of SMART, artificial intelligence (AI), and clinical CMR: A graphical overview of the relationships between artificial intelligence (AI) and its subsets, machine learning (ML) and deep learning (DL), as well as AI’s potential impact on clinical CMR through SMART techniques. AI uses machines to perform tasks usually disposed to humans and covers all ML techniques. ML specifically investigates how computers learn from data, and DL, the most popular ML technique, automatically learns important features of data. **(B)** Examples of deep learning applications to SMART CMR workflows: DL has been applied at all stages of the CMR workflow, from image acquisition to analysis. Advantages to DL applications include reductions in input needed from clinicians through automated methods, and significant reductions in time for acquisition, reconstruction, and analysis. Challenges remain in applying DL to clinical settings, include computational limits, the lengthy training required for DL networks, and the “black box” nature of DL.

For SMART to be adopted into clinical practice, the issue of lengthy reconstruction times must be addressed. Reconstruction times of SMART have shown to vary from 3 min (105) to several hours (55). However, a clinical workflow may require even faster reconstruction speeds to troubleshoot any potential issues with the acquisition that could arise while the patient is still in the scanner. The speed of reconstruction for each method depends on several factors such as acquisition parameters [dimensionality of the acquisition (2D versus 3D), in-plane spatial resolution, undersampling factor, etc.], reconstruction parameters (number of iterations and other tuneable parameters), computer hardware parameters [random access memory, processing unit (graphics processing unit vs. central processing unit), etc.], and computing platform (computed unified device architecture, python, MATLAB, etc.) (55). Deep Learning (DL) may allow SMART to overcome some of their limitations by efficiently computing complex reconstruction tasks (Figure 10).

**FIGURE 11:**
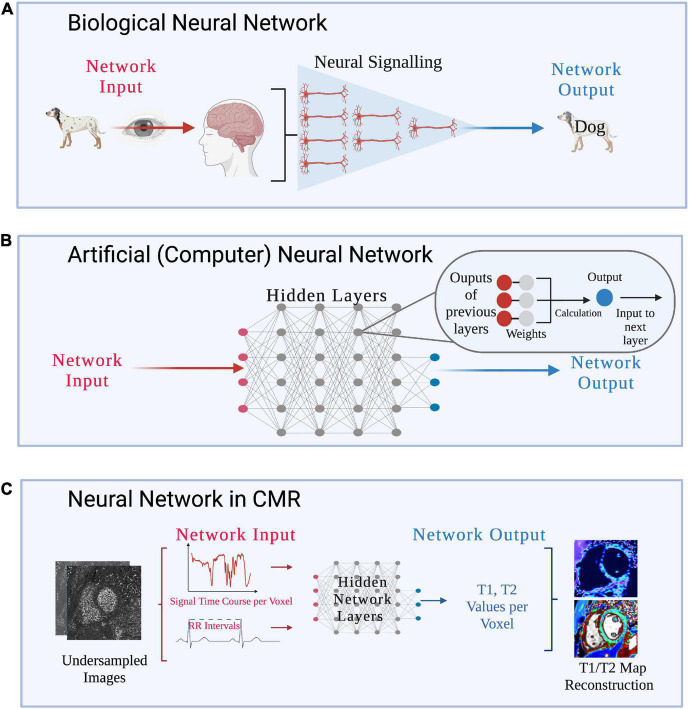
**(A)** Biological neural network: pictorial depiction of visual processing by interconnected neurons in the human brain. Figure design inspired by Zhu et al. (128). **(B)** Artificial (computer) neural network: pictorial of an artificial (computer) neural network as used in DL methods. Neural networks are composed of multiple hidden layers of interconnected nodes, which parallel the human brain’s interconnected neural signaling pathways. Network inputs go through several layers of computations which not visible to the reader, and as such, these computations are known as hidden layers. Within each layer, filters are applied to the input, producing spatially dependent features which are then input to the next layer. The network aims to learn the optimal value of the filters, known as their weight, to generate features of maximum relevance to the task. If there are many layers, or computations, in the model, it is known as a deep neural network. **(C)** Neural network in CMR: a basic graphical representation of the deep neural network structure used to accelerate cardiac Fingerprinting (cMRF), as proposed by Hamilton and Seiberlich (106). After the network has been trained on simulated cMRF data, undersampled cMRF images are input and the network produces reconstructed T1/T2 maps. Specifically, for a given voxel, the measured cMRF signal time course and cardiac RR interval times from the ECG are input. The network then produces the estimated T1 and T2 values per voxel. This technique greatly accelerates reconstruction time from undersampled images, suggesting applications for rapid CMR reconstructions in clinical settings.

Deep learning has already been applied to cMRF for optimizing the dictionary-generation, image gridding and dictionary-signal-matching process used in cMRF’s reconstruction of parametric maps (106, 107). Dictionary generation is a time-consuming and computationally heavy part of cMRF’s reconstruction (22). DL reconstructions have been shown to speed up this process by more than sixfold (106), simply outputting T1/T2 values after the MRF signal time course and cardiac RR interval times are inputted to the network (Figure 11) (107). DL has also been applied to automated planning and sequence design, useful for reducing the complexity of scanning for technologists, though limited literature exists of cardiac applications (108).

Widespread application of DL to SMART is still limited. Their novelty and their current development status limit DL to a use mostly in small, exploratory studies, rather than large clinical validation trials. In addition to the signal-dictionary matching in cMRF, DL has been applied to quickly reconstruct T1 and T2 maps directly from cMRF images (107), reconstruct feature maps from multitasking images while accelerating reconstruction time by a factor of up to 3,000 (109), accelerate the acquisition of whole-heart magnetization transfer images fivefold (28), and has accurately estimated image IQ from other sequences in line with expert human reader ratings (110). Since DL has the potential to expedite the time-consuming and computationally demanding reconstruction processes of many SMART, its application will likely increase as SMART become more mature.

The use of DL is not without limitations. While DL may offer an increase in SMART reconstruction speeds, it requires a training stage which may not be easy or fast to conduct. Computational challenges such as the limits in processing capabilities to reconstruct long image sequences (111), and the need for labeled datasets in training (111) may complicate or restrict its general utility. Small datasets used for training DL networks may not accurately represent the diversity of the true population, preventing adoption in clinical practice (111). Furthermore, the “black box” nature of DL may limit a more rapid widespread adoption (111). The performance of other sparse sampling methods (PI, CS, LRT, HD-PROST) can be characterized using mathematical tools to understand how and when these methods fail, but this is more challenging with DL because the mathematical expressions inside the neural networks are hidden. This may be especially problematic when DL methods return reconstructions that look realistic but are in-fact inaccurate descriptions of the real pathology or anatomy. Ideally, clinicians should be able to understand DL’s predictions before applying the results to clinical decision making (111). Minimizing bias in network design and ensuring training can be done with representative datasets and weighting will be critical to ensure DL networks do not simply replace manual bias with another form (109).

Despite its limitations, recent work supports the idea that SMART techniques will move toward DL reconstructions. The long manually intensive reconstructions currently experienced with SMART techniques only pushes CMR’s time limitation from the foreground to the background (42). CMR will be unable to accommodate more patients without experiencing a reconstruction backlog, but this can be solved with DL (111).

## Conclusion and future outlook

The application of SMART to clinical settings has the potential to change the current practice of CMR imaging. The ability of these techniques to acquire and then reconstruct different types of CMR images from a single image acquisition sequence simplifies the workflow for both the technologist and the patient. In the long term, this may allow CMR to be used in centers or locations without technologists specialized in cardiac imaging. The benefit may also extend to patients living in remote areas, avoiding long commutes to specialized CMR centers. The significant shortening of scan times by using SMART compared to conventional CMR sequences may allow higher patient throughputs, reducing cost per scan and shortening CMR waitlists (9). The other added benefit for clinicians is the co-registration of SMART images, as various tissue characteristics or regional function can be reliably combined and thereby better inform therapeutic decisions. As part of a comprehensive CMR exam which includes morphology, function, and tissue characterization, SMART provides opportunity to obtain several of these parameters simultaneously. Some additional technical developments and eventually large, prospective, controlled clinical trials will be required to bring these techniques into clinical routine and identify areas where the techniques need to be optimized for clinical application. However, SMART addresses the issues of complicated imaging methods and long scan times in one way or another.

## Author contributions

KE wrote and edited the manuscript, and created the figures and tables. KL assisted in drafting and editing text, and with creating figures. SR assisted in drafting text. MC assisted with idea conception and figure creation. MF assisted with text editing and is the senior author. All authors contributed to the article and approved the submitted version.
